# Sex dependency of inhibitory control functions

**DOI:** 10.1186/s13293-016-0065-y

**Published:** 2016-02-09

**Authors:** Farshad A. Mansouri, Daniel J. Fehring, Alexandra Gaillard, Shapour Jaberzadeh, Helena Parkington

**Affiliations:** Cognitive Neuroscience Laboratory, Monash Biomedicine Discovery Institute, Department of Physiology, Monash University, Victoria, 3800 Australia; ARC Centre of Excellence in Integrative Brain Function, Monash University, Victoria, Australia; Non-invasive Brain Stimulation & Neuroplasticity Laboratory, Department of Physiotherapy, Monash University, Victoria, 3800 Australia

**Keywords:** Sex dependency of cognitive functions, Executive control, Stop-signal task, Music effects, Post-error slowing

## Abstract

**Background:**

Inhibition of irrelevant responses is an important aspect of cognitive control of a goal-directed behavior. Females and males show different levels of susceptibility to neuropsychological disorders such as impulsive behavior and addiction, which might be related to differences in inhibitory brain functions.

**Methods:**

We examined the effects of ‘practice to inhibit’, as a model of rehabilitation approach, and ‘music’, as a salient contextual factor in influencing cognition, on the ability of females and males to perform a stop-signal task that required inhibition of initiated or planned responses. In go trials, the participants had to rapidly respond to a directional go cue within a limited time window. In stop trials, which were presented less frequently, a stop signal appeared immediately after the go-direction cue and the participants had to stop their responses.

**Results:**

We found a significant difference between females and males in benefiting from practice in the stop-signal task: the percentage of correct responses in the go trials increased, and the ability to inhibit responses significantly improved, after practice in females. While listening to music, females became faster but males became slower in responding to the go trials. Both females and males became slower in performing the go trials following an error in the stop trials; however, music significantly affected this post-error slowing depending on the sex. Listening to music decreased post-error slowing in females but had an opposite effect in males.

**Conclusionc:**

Here, we show a significant difference in executive control functions and their modulation by contextual factors between females and males that might have implications for the differences in their propensity for particular neuropsychological disorders and related rehabilitation approaches.

## Background

Executive control [1] is critical within everyday life as it is essential for optimizing the flexible use of limited cognitive resources to currently prioritized tasks and achieving goals [2–4]. This control might be achieved by detection of the goal-relevant information and/or suppression of irrelevant information to facilitate selection of the most appropriate behavior for achieving goals in a changing environment [1, 2, 5–8]. Response inhibition is an important aspect of executive control that deals with suppression of behaviors that are no longer appropriate or relevant [9, 10].

A prominently used neuropsychological test for assessing executive control, specifically response inhibition, is the stop-signal task [11]. Within this task, participants are instructed to perform a repetitive reaction time task (go trials), while in a small subset of trials, a stop signal will appear immediately after the onset of the go cue, and the participants are asked to withhold their responses (stop trials) [8]. The delay in the onset of the stop signal after the presentation of the go cue (stop-signal delay, SSD) is modulated to alter the probability of successful inhibition. The relationship between SSD and inhibition rate was proposed by Logan and Cowan [11] as the “horse race” model. It proposes that a go process is initiated by the presentation of the go cue and races against a stop process initiated by the stop signal, with the first process to finish determining if the initiated motor response is continued or is inhibited. Therefore, if the go process finishes before the stop process, response inhibition would fail; but if the stop process finishes before the go process, response inhibition would be successful. Imaging studies have investigated the neural substrate of these processes, particularly that of inhibitory control. It was revealed that in the stop trials, which required response inhibition, the anterior cingulate cortex (ACC) had significantly higher activation in comparison with that detected within the go trials [12]. Furthermore, Hughes et al. [13] found activation in the right inferior frontal gyrus, dorsolateral prefrontal cortex, and parietal cortex when inhibition of responses was required and reported altered activation patterns in schizophrenic patients [14] during the stop-signal task. These studies suggest that areas involved in executive control of behavior [2] support cognitive processes in the stop-signal task.

Errors in action selection might be due to task inappropriate application of executive control, such as impaired response inhibition or selection of inappropriate behavioral rule. Following an error, a cascade of autonomic, emotional, and cognitive compensatory mechanisms are initiated [15]. These mechanisms, referred to as error-induced adjustments, aim to resolve the error, optimize future behavior to reduce error likelihood, and adjust to motivation [16]. Error-induced behavioral adjustments are seen across numerous cognitive tests, such as the flanker [17], Stroop [18], and stop-signal task [10, 19]. Accompanying these modulations, a uniform trend of slowing is found in subsequent responses [20]. Previous studies [8, 11, 20] have reported that errors in inhibition of response in the stop trials results in a slowing of the response in the subsequent go trial; this phenomenon is known as post-error slowing and might reflect context-dependent adjustments in behavioral strategy (adjusting speed vs accuracy trade off).

Sex is a biological characteristic that can influence behavior [21]. Previous studies suggest that females and males show dissociable abilities in various cognitive tasks [22]. There has been significant debate regarding sex differences in executive control function, with some studies showing distinct differences [23, 24] and others showing none [25, 26]. Imaging studies have also indicated differences in regional brain activations between females and males in the context of various cognitive tasks [27, 28]. Cognitive sex differences might emerge from differences in brain network organization resulting from evolutionary trends, differences in exposure to hormones, or other developmental factors [29, 30]. These sex-specific alterations in cognitive processing might lead to alterations in preferred learning styles [31]. Sex-related differences in learning abilities have been reported in humans and non-human primates [32, 33]. In conjunction with these, response inhibition may also be influenced by sex. It has been suggested that evolutionary pressures arising from different responsibilities and constraints within groups might have led to a higher degree of self-regulation and inhibition ability in females [34, 35]. Numerous studies have revealed that males are more susceptible to impairment in inhibitory control and increased levels of impulsivity compared with females [28, 36, 37]. Impaired inhibitory control and impulsivity are hypothesized to be core deficits within substance dependence [28] and might underlie higher rates of substance use in males [21]. Schizophrenic patients show deficits in executive control tests such as stop-signal tasks, and cognitive impairments are much more exaggerated in male patients [38]. As an extension of these, it can also be suggested that, due to these innate sex differences, optimal rehabilitation treatments could be tailored for females and males.

We hypothesized that the ability to inhibit inappropriate behavior is an emerging property of executive control functions which is shaped depending on the current goals, task structure, and the dynamic of the environment. The proposed advantage of females in inhibitory control might emerge when they frequently encounter the demands and requirements in a particular task and environment. Stop-signal task is an established cognitive task that requires inhibition ability, and therefore, in the context of this task, we examined whether there were differences in inhibition ability between females and males before and/or after practicing.

Recent studies suggest that, in addition to sex, various contextual factors such as stress and uncertainty might influence cognitive functions by modulating the emotional state and mental set [39–41]. Imaging studies have shown that there is a large overlap between brain areas that are involved in organizing cognitive and executive functions and those that are activated by changes in emotional state [42]. Music is a frequently encountered salient cognitive factor [43, 44] that can potentially influence cognitive functions through its effects on mental and emotional state [45]. Previous studies have shown that music might either improve [46, 47] or reduce [48, 49] performance in perceptual, motor, or cognitive tasks. The modulatory effects of music might occur due to the influence of music over executive functions [44], as imaging studies have shown that music alters activation levels in areas responsible for executive control processes, such as the dorsolateral prefrontal cortex (DLPFC) [50]. Music might also influence emotion regulation [45], which could influence performance in cognitive tasks by altering the cognitive resources or interaction of emotional and executive control processes [51, 52]. Furthermore, it has also been highlighted that the behavioral effects of music might differ between females and males [6, 44]. Recent studies suggest that music might have beneficial effects on performance in executive control function tests [53–55]. As mentioned, schizophrenic patients show general cognitive impairments and inhibitory deficits in performing stop-signal tasks and tests of executive functions and music might have the potential to alleviate such cognitive deficits [10, 14, 56, 57].

The effects of music on cognitive functions might interact with sex. Previous studies suggest that there are differences in language processing between females and males [58, 59] which might explain males’ higher susceptibility to language-related deficits such as aphasia following the left hemisphere brain damage [59]. The significant overlap between the brain areas related to language and music processing [60–62] suggests that sex-dependent processing of auditory information might also affect music processing. Indeed, recent studies suggest that the neural correlate of music processing differs between females and males [63]. Damage to brain areas such as the superior temporal, temporoparietal, insular, and frontal cortices specifically in the right hemisphere leads to deficits in music processing and other cognitive functions [64, 65].

We hypothesized that music might have a multifaceted influence on cognitive processes. Music might act as an extra-task interfering factor and engage parts of cognitive resources and therefore adversely affect performance in ongoing tasks and at the same time directly influence the emotional state or executive control functions and exert an enhancing effect on some cognitive functions. The stop-signal task requires participation and coordination of multiple cognitive processes and is a suitable task to examine the effects of music on executive functions. Various behavioral measures in this task reflect the efficiency of the inhibitory processes and also context-dependent trial-by-trial modulations of behavior that are evoked by experiencing error (post-error slowing) or changes in task demand. It is still unclear whether and how sex and music might interact to influence executive control of a goal-directed behavior. The differential effects of music on executive control functions in females versus males have rarely been investigated. In this study, we tested female and male participants in a stop-signal task to assess their ability to inhibit planned movement as an index of executive control function. We aimed at examining how exposure to task demands and practice would affect inhibitory ability in a stop-signal task, whether it is dependent on participants’ sex and whether music would influence these processes.

## Methods

### Participants

Thirty-nine Monash University undergraduate (third year) students were recruited to perform the stop-signal task for two separate 2-h sessions 1 week apart. The 20 females (mean age 20.7 ± 0.3 years) and 19 males (mean age 21.2 ± 0.3 years) had no history of neurological disorders and joined this research project on a voluntary basis. There was no significant difference in age between the female and male participants (two-tailed *t* test, *p* = 0.34). Seven females and 6 males mentioned that they listen to music during their studies but the type of music was not specified. Approval was obtained from Monash University Human Research Ethics Committee. Written consent was obtained from all participants.

### Apparatus

An automated test apparatus was used to perform the stop-signal task. The subjects were seated in front of a touch-sensitive screen (MicroTouch surface capacitive touch display (17″)) on which the stimuli were displayed. The participant’s head distance from the monitor was about 60 cm. Although the participants were advised to gaze at the center of the monitor, no head or eye fixation was required. The size of each stimulus on the screen was between 5 and 7 cm. A switch was placed on a wrist-rest pad at the middle bottom of the monitor. The participants were advised to use the index finger of their dominant hand to press the switch and touch the items on the screen. A monitoring camera allowed the participants to be observed while performing the task. CORTEX program (National Institute of Mental Health) was used to control the experiment and data acquisition at millisecond (ms) resolution. Before performing the test, the participants read an instruction pamphlet explaining the task requirements, which was followed by brief verbal instructions. The participants were instructed to perform as fast and accurately as possible.

### Behavioral task

**Fig. 1:**
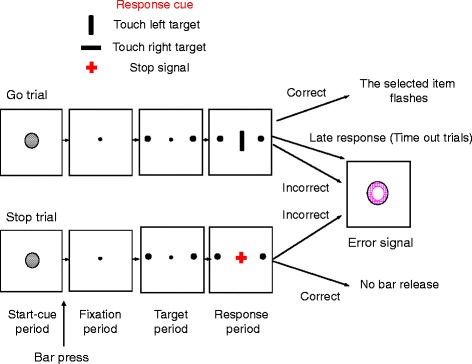
The stop-signal task. In go trials, after the onset of the start cue (gray circle), the participants had to push a switch (located at the bottom of the monitor) with the dominant hand within 10 s. The switch pressing changed the start cue to a fixation point. If the participants kept the switch pressed for 450 ms, two small targets (white circles) appeared at the left and right sides of the fixation point. If the participant maintained switch pressing for another 300 ms, the fixation point was turned off and a direction cue (horizontal or vertical white bar) was presented at the center of the screen (black background). The horizontal bar instructed touching the right-side target (right go trial) but the vertical bar instructed touching the left-side target (left go trial). The left and right go trials were presented randomly and in the same proportion. The participants had to release the switch and touch the target within a limited time window (900 ms from the onset of the response cue). Failure to touch the screen in this time window was considered as a time-out error. After correct target selection, a feedback was given to the participants (the selected target flashed twice). After an erroneous target selection or early release of the switch, all the items were turned off and a visual error signal (a purple annulus) was shown for 500 ms. Events in the stop trials were similar to those in the go trials; however, after direction-cue onset, a red cross (stop signal) replaced the direction cue. The participants had to maintain pressing the switch after seeing the stop signal. Failure to stop the response in the stop trials (switch release) was considered as an error, and the error signal was shown

Each trial began with the appearance of a start cue, which required the participants to press on a switch (Fig. 1). This was followed by the appearance of a fixation point (for 350 ms), and then by two target items (small white circles) on the left and right sides of the screen (for 300 ms). If the participants kept the switch pressed, a go-direction cue appeared at the center as a cue to initiate the response. The go-direction cue was either a horizontal or vertical white bar that instructed the right or left target selection, respectively. The participants were instructed to respond as fast as possible to the go-direction cue by releasing the switch and touching the correct target item on the screen within a time window of 900 ms. Trials requiring left or right target selection were intermingled and run randomly and in the same proportion. Failure to touch the screen in the time window was considered as an error, which led to the disappearance of all the items. Early release of the switch before the onset of the go-direction cue or selection of a wrong target (not matching the instruction given by the go-direction cue) was considered as an error which led to the disappearance of all the items and the presentation of an error signal for 500 ms.

In the stop trials, the events were the same as in the go trials; however, a stop signal (red cross) appeared with a delay after the go-direction cue and instructed the participants to inhibit their response and hold the switch pressed (Fig. 1). Stop signals occurred in 30 % of trials (with the same proportion of trials requiring left or right target selection). The go and stop trials were intermingled and shown randomly. The stop signal was initially presented 15 ms after the presentation of the direction cue (i.e., the SSD in the first trial of the stop trials was 15 ms). The SSD was adjusted depending on the participant’s performance. If the participant successfully inhibited their response, the SSD would increase by 40 ms each time. If the participant failed to inhibit their response, the SSD would remain at 15 ms or decrease by 40 ms, if the SSD had previously increased. For calculation of the percentage of correct responses in the stop trials, the first four stop trials in each session were excluded.

The first block was a practice block and comprised of only the go trials where the participants had to reach 90 % accuracy before entering the main block where the data for this study were collected. Each daily testing had two sessions, and in each session, the participants were required to complete 200 trials in the main block. There was a 10-min break between the two sessions. Each participant completed two testing held 1 week apart. In one testing session, each participant listened to music while performing the task, whereas in the other, the participant wore the same noise-canceling headphones but without music. This was counterbalanced between the first and second tests and between males and females, as in the first testing, 50 % of females and males listened to music and 50 % of females and males had silence. This was reversed in the second testing.

The response time was calculated from the onset of the go-direction cue to the release of switch (not the screen touch). Switch release was registered at millisecond resolution. In some of the previous studies of the stop-signal task, participants had to press one of two switches depending on the go-cue information and the motor responses were delivered by two hands [13] or two fingers [11]. However, any advantage in differentially using the left or right hand/finger might have affected the response time and its variation. Compared with young females, young males more frequently play computer games, which require sensory-motor coordination and therefore differential use of fingers were supposedly more perfected in males. Our task paradigm and motor response requirement were designed to minimize the possible effects of differences in using particular hand or fingers that might have been developed by differences in typing, gaming, or sport [66]. In our task, the go-direction cue instructed touching the left or right target on the screen, but the first step in this response was releasing the switch. Therefore, for all the responses, the release of switch was done by the index finger of the dominant hand and could not be affected by the advantages in using particular fingers or hands.

#### Music

We used contemporary pop music with lyrics. The songs were a mixture of low and high tempo songs with lyrics. Our main goal was to study the effects of exposure to background music, as occurs during listening to radio, on cognitive functions. The selection criterion for the songs was absence of any offensive statement in the lyrics. We set the volume for all participants, but the participants were allowed to adjust it if they found it too low or too high. The same songs were played in a random order for all participants.

### Data analyses

Data were collected from all four sessions over the two daily testings. We measured the time from the onset of the go-direction cue to the switch release as the response time (RT). By taking the mean RT and the mean stop-signal delay (SSD), we calculated the stop-signal reaction time (SSRT = mean RT − SSD). Raw data (percentage of correct responses or response time) were used in all analyses; however, to ease comparison between groups and conditions, the mean response time in each session was normalized by dividing by the grand average for all conditions.

Previous studies [8, 11, 20] have reported that errors in inhibition of response in the stop trials result in a slowing of the response in the subsequent go trial (post-error slowing). In our study, post-error slowing was examined by comparing the response time in the correct go trials that were preceded by a failed stop trial against correct go trials that were preceded by correct go trials [67].

Partial eta squared indicates the proportion of the variance explained by the effect in ANOVA and was calculated for each significant sex-dependent modulation of the behavioral measures.

## Results

In the go trials, the participants started each trial by pressing a switch, which led to the appearance of a left and right target on the screen (Fig. 1), which was followed by a go-direction cue. Upon the onset of the go-direction cue, the participants had to release the switch and touch the left or right target on the screen. In the stop trials, a stop signal appeared after the go-direction cue and instructed inhibition of the response. In the stop trials, holding the switch down was considered the correct inhibition of response (correct stop trial). However, release of the switch was counted as an error in the stop trials, and an error signal replaced all the items. An adaptive procedure was used to keep the percentage of correct responses in the stop trials around 50 %. In each session, the delay between the onset of the stop signal and go-direction cue (SSD) was adjusted depending on the success of the participants in inhibiting their response in the stop trials [11]. The participants’ percentage of correct responses in the stop trials were 54.4 ± 0.3 (mean ± SE) and 53.8 ± 0.4 in silence and music sessions, respectively, indicating that the adaptive procedure was effective in bringing the performance close to 50 %. A three-way ANOVA [sex (female/male, between-subject factor) × session (first/second, within-subject factor) × music (silence/music, within-subject factor)] was applied to the percentage of correct responses in the stop trials and showed that the main effects and the interactions between factors were not significant (*p* > 0.05).

### Learning in stop-signal task differed between females and males

**Fig 2:**
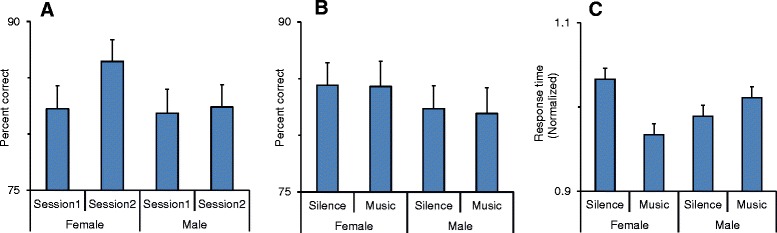
Sex-dependent modulation of behavior in stop-signal task. **a** The percentage of correct responses in different sessions are shown for male and female participants. In the second session, the percentage of correct responses significantly increased for female participants. **b** The percentage of correct responses in go trials did not significantly change as a result of listening to music. **c** The normalized response time in go trials is shown in sessions with and without music for female and male participants. Response time decreased in females but increased in males, while the participants listened to music. *Error bars* in all the figures show the standard error

We found that participants’ performance in the go trials was influenced by practice and sex. The three-way ANOVA [sex × session × music] was applied to the percentage of correct responses in the go trials. The main effect of sex was not significant (*F*(1,37) = 0.65; *p* = 0.43); however, there was a significant effect of session (*F*(1,37) = 11.19; *p* = 0.002) indicating that the percentage of correct responses increased from the first to the second session in the same testing day. There was also a significant interaction between sex and session (*F*(1,37) = 6.68; *p* = 0.014) (partial eta squared = 0.15) indicating that the improvement in performance was seen in females (Fig. 2a). The main effect of music was not significant (*F*(1,37) = 0.05; *p* = 0.48), and music had no interaction with other factors indicating that listening to music did not affect the percentage of correct responses in the go trials (Fig. 2b).

### Context-dependent adjustment of behavior was influenced by sex and music

**Fig. 3:**
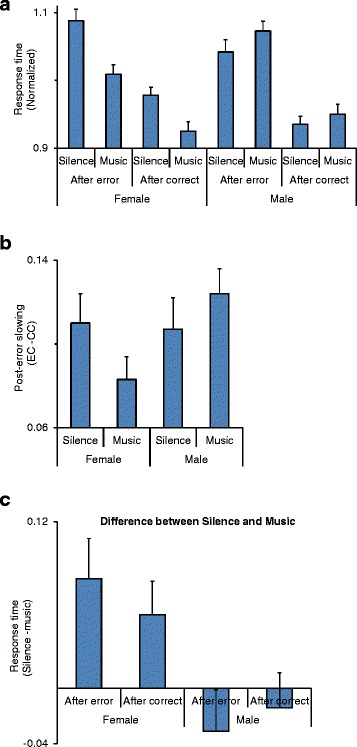
Interaction of music and error in influencing the behavior. **a** The normalized response times in after-error (EC: a correct go trial preceded an error in the stop trial) and after-correct (CC: a correct go trial preceded by a correct go trial) trials are shown in music and silent sessions for female and male participants. Response time was longer in the after-error trials in female and male participants. **b**–**c** Post-error slowing (EC–CC) was modulated by music depending on the sex of the participants. **b** While listening to music, post-error slowing decreased in females but it was increased in male participants. **c** The difference in normalized response time between silent and music sessions is shown in the after-error and after-correct trials

Previous studies [8, 11, 20] have shown that error in inhibition of response in the stop trials is followed by a slowing of the response in the subsequent go trial (post-error slowing). We examined this post-error slowing by comparing response times in those correct go trials that were preceded by failed stop trials (i.e., releasing the switch in the stop trials) (after-error: EC trials; E = Error and C = Correct) with those correct go rials that were preceded by correct go trials (after-correct: CC trials). Response time was calculated as the time between the onset of the go signal and the release of the switch. A four-way ANOVA [post-error (after-error/after-correct, within-subject factor) × session × music × sex] was applied to the response time in the second trial of EC and CC trial sequences. The main effect of the post-error was highly significant (*F*(1,37) = 204.6; *p* < 0.00001) indicating that response time increased after errors in a stop trial (Fig. 3a: the difference between the after-error and after-correct trials). The main effect of music (*F*(1,37) = 1.1; *p* = 0.30) or sex (*F*(1,37) = 3.32; *p* = 0.080) was not significant. The main effect of session (*F*(1,37) = 0.16; *p* < 0.69) or interaction of session and sex factor was not significant indicating that in both females and males, there was no significant change in response time between sessions.

There was no significant interaction between post-error and sex or between post-error and music. However, there was a significant interaction between music and sex factors (*F*(1,37) = 5.3; *p* = 0.027) (partial eta squared = 0.13) indicating that the female participants became faster but males became slower during the presence of music (Fig. 2c and Table 1). The ANOVA also showed that there was a significant interaction between post-error × music × sex (*F*(1,37) = 5.54; *p* = 0.024) (partial eta squared = 0.13) indicating that post-error slowing was modulated by music depending on the sex of the participant (Fig. 3b–c). Figure 3b shows that in both silent and music sessions and for both sexes, the response time increased in the go trial that was preceded by a failed stop trial (after error). However, music differentially affected the post-error slowing (EC–CC) depending on the sex of the participant decreasing the post-error slowing in females but increasing it in males (Fig. 3b–c). The sex-dependent effects of music on post-error slowing could not simply result from the sex-dependent effect of music on response time (Fig. 2c) because if music had evenly affected the response time in the after-error and after-correct trials, there would have been no difference in post-error slowing between silent and music sessions. However, music differentially affected the response time in the after-error and after-correct trials depending on the sex of the participants (Fig. 3b–c). Figure 3c shows that the difference between silent and music was larger in the after-error trials than after-correct trials in females but the differences were in the opposite direction in the male participants.

**Table 1:** Behavioral measures in females and males in the context of stop-signal task

	RT in go trials	RT in go trials	SSRT	SSRT	SSRT	SSRT
	Silent	Music	Session 1	Session 2	Silent	Music
Female	446.07 ± 13.32	418.26 ± 13.51	206.99 ± 7.51	191.58 ± 6.31	197.52 ± 7.24	201.05 ± 7.48
Male	458.53 ± 13.66	468.94 ± 13.86	207.10 ± 7.70	208.37 ± 6.47	204.80 ± 7.43	210.67 ± 7.67

The two leftmost columns show the response time (RT) in females and males. The other four columns show the stop-signal reaction time (SSRT) in females and males

**Fig. 4:**
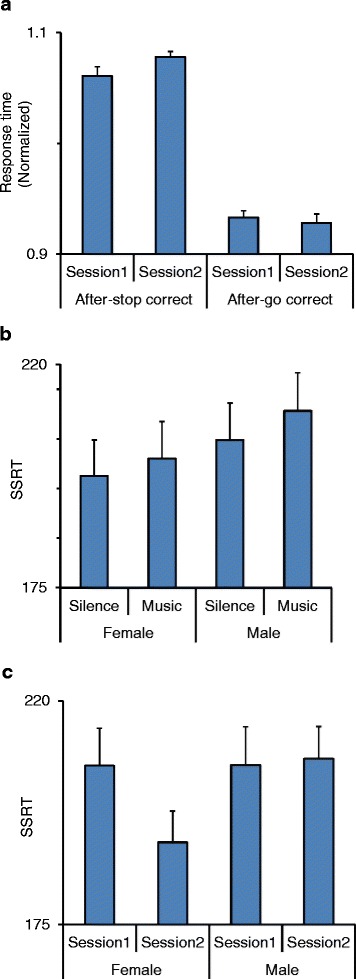
Effects of practice and music on stop-signal reaction time (SSRT). **a** Response time decreased in the go trials that were preceded by the stop trials. Normalized response time is shown in consecutive sessions in after-stop (a correct go trial that was preceded by a correct stop trial) and after-go trials (a correct go trial that was preceded by a correct go trial). In both sessions, response time decreased after a correct stop trial. **b**–**c** SSRT was calculated as the difference between the mean response time in the correct go trials and the mean of stop-signal delays (the delay between the onsets of stop signal and go-direction cue). **b** In both females and males, there was no significant change in SSRT while the participants listened to music. **c** SSRT is shown in the first and second sessions. SSRT in the second session decreased in females, but not in males, indicating that practice decreased SSRT in females but not in males

We also compared response time between those correct go trials that were preceded by a successful stop trial (i.e., not releasing the switch in the stop trials) (CsCg trials) with those correct go trials that were preceded by correct go trials (CgCg trials). A four-way ANOVA [post-stop (after-stop/after-go, within-subject factor) × session × music × sex] was applied to the response time in the second trial of CsCg and CgCg pairings. The main effect of post-stop was highly significant (*F*(1,37) = 357.08; *p* < 0.00001) indicating that the response time significantly increased in the go trials that followed a successful inhibition of response in the stop trials (Fig. 4a). There was a significant interaction between post-stop and session factors (*F*(1,37) = 8.04; *p* = 0.007) indicating that after practice, the response time increased in the after-stop trials but decreased after the go trials (Fig. 4a) and led to an increase in post-stop slowing (the difference between CsCg and CgCg) in the second session. However, the main effect of music (*F*(1,37) = 1.62; *p* = 0.21) or session (*F*(1,37) = 0.81; *p* = 0.37) was not significant and there was no significant interaction between post-stop × music × sex (*F*(1,37) = 1.22; *p* = 0.28) or between other factors.

### SSRT as an index of inhibitory function differed between females and males

We calculated the stop-signal reaction time as a difference between the mean response time and the mean stop-signal delay [11]. Figure 4b shows the SSRT in the female and male subjects in music and silent sessions. A three-way ANOVA [session × music × sex] applied to the SSRT showed that the main effect of music (*F*(1,37) = 0.80; *p* = 0.38) or session (*F*(1,37) = 3.31; *p* = 0.077) or sex (*F*(1,37) = 0.85; *p* = 0.36) was not significant, and there was no significant interaction between music × sex (*F*(1,37) = 0.05; *p* = 0.83) (Fig. 4b). However, there was a significant interaction between session and sex (*F*(1,37) = 4.6; *p* = 0.038) (partial eta squared = 0.11) (Fig. 4c) indicating that SSRT in the second session decreased in females but not in males. A lower SSRT indicates a better ability in inhibition of responses and therefore the shorter SSRT in the second session of task suggests that in females, but not in males, inhibition ability improved by practice.

## Discussion

Our findings identified intriguing differences between females and males in learning from practice and in the influence of background music in the context of the stop-signal task. We will discuss the significance of these findings in two main parts: (1) learning from practice and (2) the effects of music.

### Learning from practice in stop-signal task differed between female and male participants

The adaptive procedure in the stop trials adjusted the difficulty of response inhibition depending on the participants’ performance, and therefore, the performance of the participants in the stop trials was kept around 50 %. This enabled calculation of SSRT as an index of inhibitory functions [8, 11]. We found significant differences in learning from practice between female and male participants.

#### Percentage of correct responses in the go trials

There was no significant difference in the percentage of correct responses between female and male participants before practice (Fig. 2a). The percentage of correct responses in the second stage of testing in each daily session was significantly larger in the female participants indicating that the rate of learning was much faster in females. Go trials required rapid selection of the target based on the go-direction cue (which instructed response direction) in a limited time window. However, the participants also had to be vigilant about the stop-signal appearance and the necessity for stopping the response. Previous studies [68] indicated that participants take a strategy to balance accuracy with response time to optimize their behavior dependent upon instruction. In our task, all participants had been required to optimize both accuracy and response time. The limited time window for responding and the requirement for touching the screen after releasing the bar forced the participants to maintain a high speed in their responses. We found that the female participants benefited from practice and outperformed males in the go trials.

The sex-dependent learning difference in the go trials cannot be explained by differences in muscle mass or advantages in using particular hand or fingers. In previous studies involving stop-signal tasks, the participants had to use different fingers for responding, where the identity of the instruction cue dictated using a particular finger [11] or hand [13] for each response. In our task design, the go-direction cue instructed switch release and then touching the target (left or right) on the screen and the response time was calculated as the duration between the go-direction cue onset and switch release. Therefore, the initial movement (switch release) could not be affected by the preferential use of a particular finger or hand. In addition, the accuracy or response time did not differ between females and males in the first session. Therefore, the initial movement could not be affected by non-specific factors such as better sensory-motor coordination resulting from differences in typing, game playing, or sport [69].

#### SSRT in the stop trials

SSRT reflects the efficiency of the inhibitory function in stopping the planned movement and therefore is an index of executive control function [10, 70]. A smaller SSRT indicates a better ability in inhibition of responses and a more efficient executive control of the task. There was no significant difference in SSRT between the female and male participants indicating that, before practice, the inhibitory function was comparable in both sexes (Fig. 4c; session 1). However, the SSRT in the second stage of testing in each daily session was significantly smaller in the female participants (Fig. 4c; session 2) indicating that in females, but not in males, inhibition ability significantly improved with practice. This finding suggests that practice in the stop-signal task significantly enhanced the executive control of behavior in females but did not have such an effect in males. SSRT is calculated as the difference between the mean SSD and the mean response time and is independent of differences in participants’ response time [11]. Our results showed that in both females and males, there was no significant change in response time by practice. Therefore, the enhanced ability of the female participants in inhibiting the responses could not be related to differences in response times.

These results show that after practice, the female participants significantly improve their performance in the go trials (increased percentage of correct responses) and also in the stop trials (shorter SSRT). Our findings indicate a superb ability in females in learning from practice and improving executive control function. Li et al. [26, 28] used functional magnetic resonance imaging (MRI) to examine neural activation in female and male participants performing a stop-signal task. Although there was no significant difference in behavioral measures between the two sexes, the activation patterns differed between females and males [26, 28] suggesting that females and males use different neural processes to control task performance in stop-signal tasks. Such differences might be related to fundamental differences in executive control adjustments between females and males that lead to their known differences in susceptibility to compulsive behavior and drug addiction [36, 37, 71]. In contrast to our results, Li et al. [67] did not find any significant difference in behavioral measures between females and males. There are differences in the task paradigm and in the number of completed trials between the Li et al. [67] study and ours. In Li et al. [67] study, each participant performed about 315 go and 105 stop trials, but in our study, the participants performed a total of 800 trials (about 560 go and 240 stop trials). In our study, all the participants were third year undergraduate students, and therefore, the cohort was very uniform in terms of age and education level. In the Li et al. [67] study, the age range (22–40) and possibly education were much broader. Therefore, in our study, with more trials and a more uniform participant cohort, the power of analyses might have been higher in the potential to detect differences. In Li et al. [67] study, the go cue required a single response (button press) but in our study, the go cue not only instructed the initiation of response but also informed about the required response direction (vertical and horizontal bars instructed left and right side response, respectively), and therefore, the participants had to map this direction information into their delivered response. This might have added to the cognitive demand in the course of executing-inhibiting responses. In contrast to our study, Li et al. [67] did not report on the practice effect, and therefore, it is unclear whether there was a practice-related change in behavioral measures (and activation pattern). Indeed, considering our findings, it would be important to investigate whether there are sex-dependent neural activations correlated with practice-related changes in behavioral measures.

It has been proposed that evolutionary pressures have led to a greater capacity of females in inhibition of inappropriate responses leading to a higher chance of finding the appropriate mate and protecting the family [72]. This proposal has been supported by some studies showing that females, even at a young age, show more self-restraint and inhibition in social and cognitive behavior; however, conflicting results in other studies have left this issue unresolved [35]. Our findings indicate that before practice, there was no significant difference in SSRT between females and males; however, a significant difference emerged after practicing inhibition in a stop-signal task. The ability to learn from practicing inhibition might be a crucial dissociating factor that provides advantage for females in adapting to their environment and task demands. Previous studies have suggested that females show better outcomes after rehabilitation and abstinence from drug abuse, which presumably requires sustained inhibition of compulsive drug seeking and abuse behavior [73]. Our study, in a more controlled environment, shows that females significantly enhance their inhibition ability after practice.

### Effects of music on behavioral measures in stop-signal task

The participants performed the stop-signal task in silence or while listening to background music, and we found that music affected behavioral measures in the stop-signal task.

#### Percentage of correct responses

The percentage of correct responses in the first and second sessions were not influenced by listening to music (Fig. 2b). Similarly, learning from practice, which appeared as improved performance in the second session, was not affected by music.

#### Response time

Music had a significant effect on response time, depending on sex (Fig. 2c). In the go trials, females became faster but males became slower while listening to music. This finding suggests that music enhanced speed of target selection in females but had an opposite effect on males’ response. This sex-dependent effect of music could not result from differences in music type because all the participants listened to the same set of songs.

#### Post-error slowing

After commission of an error in inhibiting the response in the stop trials, the response time was significantly longer in the following go trials (Fig. 3a). The participants had been instructed to maximize both accuracy and response speed. However, after commission of an error in the stop trials, there might have been an adjustment in behavioral strategy (adjusting speed-accuracy trade-off) to decrease the error likelihood [8]. These behavioral adjustments might be mediated by proactive control. Proactive control is the restraint of actions seen when an error or other contexts are anticipated [74]. This occurs within the stop-signal task, as participants anticipate stop signals; therefore, after an error, they are more hesitant with their responses [74, 75]. Imaging studies have shown activation of supplementary motor cortex and midbrain areas when the necessity for inhibition is anticipated [74]. We found a significant interaction between post-error slowing and music and participants’ sex (Fig. 3b-c). Post-error slowing while listening to music was attenuated in females but became larger in males. The sex-dependent modulatory effect of music on post-error slowing cannot be explained simply by the enhancing effect of music on females’ response time. If the response time of female participants in the second trial of EC (after error) and CC (after correct) trial sequences was enhanced to the same proportion by music, then the post-error slowing (EC–CC) would have remained unchanged. But the post-error slowing decreased in females while listening to music, which indicates that the effect of music was larger in EC trials (Fig. 3c). These findings suggest that the effects of music were dependent on the history of error commission and were also sex dependent. Li et al. [67] reported activation changes mainly in the right ventral-lateral prefrontal and also in the right middle frontal and fronto-polar cortices in relation to the post-error slowing. A recent study in non-human primates also showed that the fronto-polar cortex might be involved in the context-dependent executive control adjustments [76]. The sex dependency of the music effect on post-error slowing might be related to sex differences in functional organization of neural networks that involve the prefrontal cortex [28, 67, 77, 78].

#### Post-stop slowing

The response time was significantly slower in those trials that followed a successful inhibition of response in the stop trials. This indicates that after failure or success in inhibiting the response in the stop trial, the participants’ response time increased in the following go trial and appeared as post-error slowing or post-stop slowing, respectively. This after stop trial slowing might reflect a momentary shift of behavioral strategy to increase accuracy in the following trial by lengthening the response time. The post-stop slowing was significantly enhanced by practice which suggests that after practice, the participants were more inclined in adapting this strategy. However, the post-stop slowing was not different between females and males and was not affected by listening to music. These findings indicate that the sex dependency of post-error slowing under the music effect was not related to the trial type (stop trial) in the preceding trial but was dependent on whether an error occurred in inhibiting the response in the preceding stop trial. Li et al. [67] found differences in neural activation pattern between post-stop and post-error behavioral adjustments, which suggest that post-stop and post-error behavioral modulations have different neural substrates and underlying mechanisms and therefore were differentially influenced by music in our study.

#### How music might influence executive control function?

Our study aimed at examining the effects of background music in the context of a cognitive task requiring executive control functions and showed that, indeed, listening to music had different consequences in females and males and specifically affected response time and error-induced behavioral adjustments. Music is a salient cognitive factor, and previous studies have shown that background music can either improve [47] or reduce cognitive performance [49]. These effects might be mediated through alterations in executive control function [43, 44]. Music modulates activation in the DLPFC and anterior cingulate cortex (ACC) [51, 79] and therefore might influence executive control. A link has been found between the brain regions responsible for executive control and brain regions involved in emotional regulation [51]. This is relevant to music, as music has been found to influence emotional regulation [45]. Lastly, the DLPFC also exhibited the same trend, as it was significantly activated in music making or when listening to favored music [45]. It has been found that during listening to music, men are more distractible and commit more errors compared to women, suggesting that music and auditory information might sex-dependently influence the attentional resources [44]. Together, these studies show that music acts as a context and induces behavioral adjustment possibly through influencing executive control function [52]. Alterations in ACC activation are mainly seen when music was favored or the participant had an active role in the production of the music [79]. Our main goal was to study the effects of exposure to background music, as occurs during listening to radio or in public places, on cognitive functions. We did not select the songs based on the participants’ preference. Preference needs to be subjectively rated for each song and the rating itself might differ between females and males and appear as a confounding factor and mask the existing differences between females and males. We also did not examine the effects of music type because it could vary in many different aspects (low/high tempo; with or without lyrics; sad/happy; old/new). However, our results indicate that the same type of background music sex-dependently influence cognitive functions, and future studies would examine whether these modulations depend on the music type.

## Conclusions

We examined the effects of ‘practice to inhibit’ as a model of rehabilitation approach and ‘music’ as a potential contextual factor in influencing cognition, on the ability of females and males to perform the stop-signal task. Our findings identified a significant difference between females and males in learning when they practice inhibition of inappropriate behavior. Females showed superb capability in benefiting from prior exposure and practice in using executive control in challenging tasks. This might have important implication in strategies and rehabilitation programs for controlling compulsive behavior and improving cognitive function in neuropsychological disorders. Our study also showed that music sex-dependently influenced response time. These findings suggest that music affects executive control functions and therefore could potentially be used for enriching rehabilitation and management of compulsive behavior or neuropsychological disorders.
